# Implementation of ask-advise-connect for smoking cessation in Dutch general practice during the COVID-19 pandemic: a mixed-methods evaluation using the CFIR framework

**DOI:** 10.1186/s13011-023-00535-0

**Published:** 2023-05-09

**Authors:** Naomi A. van Westen-Lagerweij, Marc C. Willemsen, Esther A. Croes, Niels H. Chavannes, Eline Meijer

**Affiliations:** 1 The Netherlands Expertise Centre for Tobacco Control, Trimbos Institute, PO Box 725, 3500 AS Utrecht, The Netherlands; 2 Department of Health Promotion, Maastricht University, PO Box 616, 6200 MD Maastricht, The Netherlands; 3 Public Health and Primary Care, Leiden University Medical Center, PO Box 9600, 2300 RC Leiden, The Netherlands; 4 National eHealth Living Lab, PO Box 9600, 2300 RC Leiden, The Netherlands

**Keywords:** Ask-Advise-Connect, Implementation, General practice, COVID-19 pandemic, Mixed-methods

## Abstract

**Background:**

The Ask-Advise-Connect (AAC) approach can help primary care providers to increase the number of people who attempt to quit smoking and enrol into cessation counselling. We implemented AAC in Dutch general practice during the COVID-19 pandemic. In this study we describe how AAC was received in Dutch general practice and assess which factors played a role in the implementation.

**Methods:**

A mixed-methods approach was used to evaluate the implementation of AAC. Implementation took place between late 2020 and early 2022 among 106 Dutch primary care providers (general practitioners (GPs), practice nurses and doctor’s assistants). Quantitative and qualitative data were collected through four online questionnaires. A descriptive analysis was conducted on the quantitative data. The qualitative data (consisting of answers to open-ended questions) were inductively analysed using axial codes. The Consolidated Framework for Implementation Research was used to structure and interpret findings.

**Results:**

During the study, most participants felt motivated (84–92%) and able (80–94%) to apply AAC. At the end of the study, most participants reported that the AAC approach is easy to apply (89%) and provides advantages (74%). Routine implementation of the approach was, however, experienced to be difficult. More GPs (30–48%) experienced barriers in the implementation compared to practice nurses and doctor’s assistants (7–9%). The qualitative analysis showed that especially external factors, such as a lack of time or priority to discuss smoking due to the COVID-19 pandemic, negatively influenced implementation of AAC.

**Conclusions:**

Although AAC was mostly positively received in Dutch general practice, implementation turned out to be challenging, especially for GPs. Lack of time to discuss smoking was a major barrier in the implementation. Future efforts should focus on providing additional implementation support to GPs, for example with the use of e-health.

**Supplementary Information:**

The online version contains supplementary material available at 10.1186/s13011-023-00535-0.

## Background

Each year, smoking tobacco accounts for approximately 7.7 million deaths and 200 million disability-adjusted life-years worldwide [1]. Stimulating people to quit smoking and offering assistance in quitting is necessary to reduce the high mortality and morbidity of smoking-related disease [2]. The importance of smoking cessation has recently received more attention due to the evidence that people who smoke have an increased risk of developing severe COVID-19 [3]. Quitting smoking has, however, been challenging for many people during the COVID-19 pandemic. While some people who smoke decreased their tobacco use during the COVID-19 pandemic, others maintained or even increased their use of tobacco [4]. Research also found that fewer people tried to quit smoking during the pandemic and that people who smoke were less successful at quitting compared to before the pandemic [5, 6]. These findings emphasize the need for efforts to increase successful quit attempts, especially in turbulent times, such as the COVID-19 pandemic.

A quit attempt is most likely to be successful when evidence-based cessation assistance is used, such as behavioural counselling and pharmacotherapy [7, 8]. Healthcare professionals can play an important role in identifying patients who smoke, stimulating quit attempts and increasing the use of evidence-based support. They can do this by providing a quit advice and offering assistance to all patients who smoke [9], and by proactively referring motivated patients to a smoking cessation program [10]. Proactively referring patients means that healthcare professionals actively connect the patient to a cessation program, for example by directly scheduling an appointment for the patient with a counsellor or by forwarding the patient’s contact details to a cessation program which in turn contacts the patient. Proactive referrals result in higher treatment enrolment rates compared to passive referrals, which require patients to contact a counsellor or cessation program on their own [10].

The Ask-Advise-Connect (AAC) approach is a brief and effective method which includes the abovementioned steps (i.e., asking patients about tobacco use, advising all patients who smoke to quit, and proactively referring patients who smoke to counselling) [11]. Although the feasibility and effectiveness of AAC has already been studied in several healthcare settings [11–15], only a few studies have investigated which strategies are needed to successfully implement AAC in practice [16, 17]. Specifically in stressful times, a comprehensive implementation strategy may be needed to implement AAC in practice.

We implemented AAC for smoking cessation within Dutch general practice during the COVID-19 pandemic by using a comprehensive implementation strategy (described in the ‘ Methods’ section). Originally, AAC was designed to directly connect patients to cessation treatment of telephone quitlines through an automated link within the electronic health record (EHR). [11] In the Netherlands, however, public telephone quitlines for cessation treatment do not exist. Instead general practice plays a central role in providing smoking cessation care to patients. As of 2019, indicated prevention is officially seen as a ‘core task’ of the Dutch general practitioner (GP) [18]. This means that GPs are responsible for discussing risk factors such as smoking with patients and preventing (complications of) chronic diseases among patients by offering support to quit. As GPs often do not have enough time to provide smoking cessation counselling themselves, they typically delegate this task to a trained practice nurse (PN) or doctor's assistant (DA) who works under supervision of the GP [19]. Patients can also be referred to a cessation program outside general practice, for example if more specialised addiction care is required or if the patient wants to receive group therapy. In 2022, 18.9% of the adult population in the Netherlands smoked, and each year only around 5% receives cessation counselling when attempting to quit smoking [20, 21]. Therefore, implementing AAC within Dutch general practice may help to ensure that more people who smoke enrol into cessation counselling.

The present study describes how AAC was received in Dutch general practice during the COVID-19 pandemic and assesses which factors played a role in the implementation. We used the Consolidated Framework for Implementation Research (CFIR) to guide the assessment [22]. CFIR is one of the most commonly used frameworks in implementation science, and can be used to assess contextual factors which influence implementation [22]. CFIR provides an overview of 48 constructs organized into five domains: innovation (i.e., attributes of AAC, for example its perceived ease of use and advantages), outer setting (i.e., the social and political context in which AAC is implemented), inner setting (i.e., aspects of the organisation in which AAC is implemented), characteristics of individuals involved (i.e., the needs, capabilities, motivation, and opportunities of the primary care providers who implement AAC), and the implementation process (i.e., approaches used in different stages to implement AAC, and their outcomes) [22].

## Methods

### Study design and participants

We used a mixed-methods approach to describe how AAC was received in general practice and assess which factors played a role in the implementation of AAC. The implementation of AAC among 106 Dutch primary care providers took place within the context of a pre-post study between late 2020 and early 2022. Participants were employed in general practice as a GP, PN or DA, and all voluntarily participated in a Pharmaceutical Therapeutic Audit Meeting (PTAM) group (in Dutch: ‘FTO’ group). PTAM groups are existing local collaborations of around 12 primary care providers, and these groups come together several times per year to discuss and agree on the implementation of various clinical guidelines.

We approached primary care providers for participation through different recruitment channels, such as newsletters directed at PTAM groups, e-mails sent directly to contact persons of PTAM groups, and newsletters of professional associations. PTAM groups interested in participating first received information on the study procedure, data protection and data anonymisation. Each participant of a PTAM group then signed informed consent before inclusion in the study. The first PTAM group enrolled into the study late 2020. We continued recruiting PTAM groups until mid-2021. For the pre-post study design, it was not necessary for all PTAM groups to begin at the same time.

Study participation lasted nine months. Participants first delivered smoking cessation care as usual (pre-implementation). We developed a comprehensive implementation strategy which came into effect after three months of participation. During a first PTAM, participants were educated about AAC and made agreements on the delivery of AAC. Participants also received a desk card as a physical reminder, and access to online educational materials. Participants reflected on the implementation of AAC during a second PTAM after six months of participation. Study participation ended after nine months. At the end of the study, all participants received €50.

### Data collection and measures

**Fig. 1:**
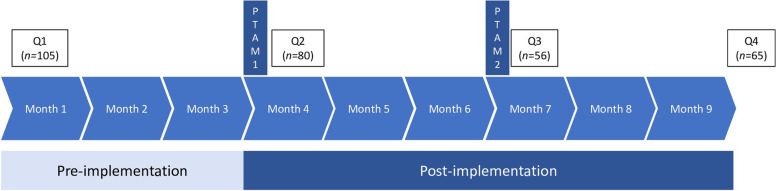
Timeline of the study, including the number of participants per questionnaire

Quantitative and qualitative data were collected during the nine months of study participation. For the current study, we used notes on the experiences of participants with implementing AAC which were taken during the two PTAMs by the first author. We also used the self-reported quantitative and qualitative data that were collected through four online questionnaires: a baseline questionnaire was sent to the participants at the beginning of the study (Q1), followed by questionnaires after the first and second PTAM (Q2 and Q3), and a final questionnaire at the end of the study (Q4). Figure 1 shows the timeline of the study, including how many participants completed each questionnaire.

The questionnaires included questions about smoking cessation care in general and the perceived influence of COVID-19, as well as perceptions of AAC and participants’ experiences with applying the AAC approach. All four questionnaires included both closed-ended and open-ended questions. Open-ended questions were answered in an open text field. The questionnaire items are described below and in Table 1.

**Table 1:** Overview of the survey questions and at which point they were measured during study participation

Theme	Closed-ended question	Open-ended question	Measured after
Changes in smoking cessation care	Did anything change with regard to smoking cessation care in your practice within the last three months, apart from the COVID-19 pandemic?	If yes: What changed?	- 3 months (Q2) - 6 months (Q3)
Influence of COVID-19 on smoking cessation care	Does the COVID-19 pandemic currently affect smoking cessation care in your practice?	If yes: How is smoking cessation care currently affected?	Baseline (Q1)
	Did the COVID-19 pandemic affect smoking cessation care in your practice within the last three months?	If yes: How did it affect smoking cessation care?	- 3 months (Q2) - 6 months (Q3)
Self-efficacy with regard to AAC	Please indicate to what extent you agree with the following statements: - I feel able to ask patients about smoking. (Ask) - I feel able to advise patients who smoke to quit. (Advise) - I feel able to proactively refer patients who smoke. (Connect)	*n/a*	3 months (Q2)
Motivation with regard to AAC	Please indicate to what extent you agree with the following statements: - I feel motivated to ask patients about smoking. (Ask) - I feel motivated to advise patients who smoke to quit. (Advise) - I feel motivated to proactively refer patients who smoke. (Connect)	*n/a*	3 months (Q2)
Expectation with regard to AAC	Please indicate to what extent you agree with the following statements: - I expect patients to react positively when I ask them about smoking. (Ask) - I expect patients who smoke to react positively when I advise them to quit. (Advise) - I expect patients who smoke to react positively when I proactively refer them. (Connect)	*n/a*	3 months (Q2)
Belief with regard to AAC	Please indicate to what extent you agree with the following statement: “I think Ask-Advise-Connect is a good method to reach patients with smoking cessation counselling.”	Please explain your answer	9 months (Q4)
Compliance with AAC agreements	n/a	To what extent are the agreements on Ask-Advise-Connect from the first PTAM complied with by you and your colleagues?	6 months (Q3)
Barriers with regard to AAC	Have you experienced any barriers in applying Ask-Advise-Connect within the last three months?	If yes: which barriers?	6 months (Q3)
	Are there any barriers which you currently encounter when applying Ask-Advise-Connect?	If yes: which barriers?	9 months (Q4)
(Dis)advantages with regard to AAC	Do you think applying Ask-Advise-Connect provides advantages?	If yes: which advantages?	9 months (Q4)
	Do you think applying Ask-Advise-Connect provides disadvantages?	If yes: which disadvantages?	9 months (Q4)
Ease of use with regard to AAC	Is it more common that Ask-Advise-Connect is easy or difficult for you to implement?	*n/a*	9 months (Q4)

#### Changes in smoking cessation care

After the first and second PTAM, participants indicated, with ‘yes’ or ‘no’, whether anything had changed with regard to smoking cessation care in their practice within the last three months, apart from the COVID-19 pandemic. Those who answered ‘yes’ were asked to describe what had changed.

#### Self-efficacy, motivation, expectations and beliefs with regard to AAC

After the first PTAM, participants indicated on a 5-point Likert scale whether they felt able (i.e., self-efficacy) and motivated to apply each step of the AAC approach, and whether they expected patients to react positively to each step of the AAC approach. For nine statements (e.g., “I feel able to ask patients about smoking”), participants could choose between ‘completely disagree’, ‘disagree’, ‘neutral’, ‘agree’ and ‘completely agree’. In the final questionnaire, participants were asked to indicate on a 5-point Likert scale whether they ‘completely disagree’, ‘disagree’, were ‘neutral’, ‘agree’ or ‘completely agree’ with the following statement: “I think Ask-Advise-Connect is a good method to reach patients with smoking cessation counselling.” Participants were also asked to explain their answer.

#### Compliance with the AAC agreements

After the second PTAM, participants were asked to describe the extent to which they and their colleagues complied with the agreements on AAC made during the first PTAM.

#### Barriers, (dis)advantages and ease of use with regard to AAC

After the second PTAM, participants indicated, with ‘yes’ or ‘no’, whether they had experienced any barriers in applying AAC within the last three months, and if so, which barriers. In the final questionnaire, participants indicated, with ‘yes’ or ‘no’, whether they currently encounter barriers when applying AAC, and if so, which barriers. Furthermore, participants indicated, with ‘yes’ or ‘no’, whether AAC provides advantages/disadvantages, and if so, which advantages/disadvantages. And lastly, participants were asked whether AAC is more often easy or more often difficult to implement. They could choose between ‘more often easy than difficult’ and ‘more often difficult than easy’.

#### Influence of COVID-19 on smoking cessation care

At baseline, participants indicated, with ‘yes’ or ‘no’, whether the COVID-19 pandemic currently affected smoking cessation care in their practice, and if so, were asked to describe how. After both the first and second PTAM, participants were asked the same question with regard to the last three months.

## Analysis

A descriptive analysis was conducted using the quantitative data (i.e., the answers to the closed-end questions in the questionnaires). We computed percentages for all answer categories of each question, stratified by profession (total, GP, PN/DA). A qualitative analysis was performed by the first author using the notes from the PTAMs and the answers to the open-ended questions in the questionnaires; the analysis was checked by the last author. The notes from the PTAMs were summarized, after which key points were identified. The answers to the open-ended questions in the questionnaires were analysed using axial codes to categorize the answers. The axial codes were continuously refined during the analysis, until we arrived at the final categories which we considered to be the factors that played a role in the implementation of AAC. For each factor, we indicated whether it appeared to act as a barrier or facilitator to implementation. In the final step of the analysis, we connected each factor to a domain and construct of the CFIR framework. The analysis was thus mainly inductive, with the CFIR framework being used to structure and interpret findings.

## Results

### General findings

Ten PTAM groups, with a total of 64 GPs and 42 PNs/DAs, participated in our study. Most PNs and DAs in our study were responsible for providing smoking cessation counselling in their practice. An overview of the characteristics of the participants and their general practice can be found in Table 2. Most participants were female (82%) and worked as a GP (60%). The majority of the participants (73%) indicated that they never or sometimes apply smoking cessation care as outlined in a clinical guideline with patients who smoke. Differences in characteristics between participants who completed at least one of the three follow-up questionnaires (i.e. Q2, Q3 or Q4) and participants who completed none of the three follow-up questionnaires are presented in Supplementary Table 1 (see Additional File 1).

**Table 2:** Characteristics of the participants and their general practice at baseline (*N* = 105)^a^

Variable	Category	n (%) / mean (SD)
Age		45.3 (9.2)
Gender	Male	19 (18)
	Female	86 (82)
Profession	General practitioner	63 (60)
	Practice nurse	36 (34)
	Doctor’s assistant	6 (6)
Smoking status	Smoker	2 (2)
	Non-smoker	103 (98)
Socioeconomic position of patients	Mostly low	6 (6)
	Mostly middle	36 (34)
	Mostly high	4 (4)
	Mixed	52 (50)
	Don’t know	7 (7)
Received training in smoking cessation care	Yes	59 (56)
	No	46 (44)
Applies smoking cessation guideline with patients who smoke	Never	44 (42)
	Sometimes	33 (31)
	Often	19 (18)
	(Almost) always	9 (9)
Attention in practice for smoking cessation	Almost no attention	3 (3)
	Some attention	58 (55)
	A lot of attention	44 (42)

^a^Although 106 participants were included in the study, one participant did not complete the baseline questionnaire and therefore only the characteristics of 105 participants are presented here

Based on our notes from the PTAMs, we observed that while AAC was mostly perceived to be relevant and helpful, applying AAC was also challenging at times due to several barriers which participants encountered in practice. The GPs in particular indicated that applying the first step of ‘Ask’ was not always feasible, due to for example a lack of time (often caused by the COVID-19 pandemic). In one PTAM group there was a discussion about whether ‘Ask’ should remain the responsibility of the GP, or whether other methods for identifying patients who smoke should be used which do not involve the GP.

The quantitative analysis showed that at six months of participation (i.e., after the second PTAM), 29% of the participants (*n* = 56) had experienced one or more barriers in applying AAC. This was 48% among GPs and 7% among PNs/DAs. At the end of the study, still 20% of the participants (*n* = 65) experienced barriers in applying AAC; this was 30% among GPs and 9% among PNs/DAs. Table 3 provides an overview of the identified factors which acted as barriers and facilitators in the implementation of AAC, based on participants’ answers to the open-ended questions in the questionnaires. Table 3 shows that the most frequently mentioned barriers were related to the CFIR construct ‘critical incidents’ (within the domain ‘Outer setting’) as a result of COVID-19. The different barriers and facilitators to implementation of ACC, categorized under the five CFIR domains, will be discussed further below.

**Table 3:** Overview of factors which played a role in the implementation of AAC, based on participants’ answers to the open-ended questions in the questionnaires

CFIR domain	CFIR construct	Factor	Mentioned by
Innovation	Evidence-base	( +) AAC is scientifically proven^a^	GP
	Relative advantage	***(* +*) AAC makes it easier to discuss smoking cessation and provide advice***	GP and PN/DA
		( +) AAC results in more active provision of smoking cessation care	GP
		( +) AAC results in improved communication towards patient	GP and PN/DA
		( +) AAC results in more patients who enrol into counselling	GP and PN/DA
		(-) Not better than other methods^a^	GP
	Complexity	***(* +*) AAC is convenient and simple***	GP and PN/DA
		***(* +*) AAC can be quickly applied***	GP and PN/DA
		( +) AAC provides structure	GP and PN/DA
		(-) ‘Connect’ takes more time and can be more difficult to carry out	GP
Outer setting	Critical incidents	***(* +*) More people motivated to quit smoking due to COVID-19***	GP and PN/DA
		( +) Easier to discuss smoking due to COVID-19	GP
		***(-) Lack of time/priority in practice to discuss smoking (due to COVID-19)***	GP and PN/DA
		***(-) Consultations more often by telephone due to COVID-19***	GP and PN/DA
		***(-) Fewer patients seen due to COVID-19***	GP and PN/DA
		***(-) Patients less motivated to quit or they delay quit attempt due to COVID-19***	GP and PN/DA
		(-) Problems with availability of smoking cessation medication	GP
	Local attitudes of innovation recipients	( +) AAC offers patient advantages, such as not needing to make an appointment themselves	GP
		(-) AAC is not always received well by patients	GP
	Partnerships & connections	(-) Lack of group counselling nearby	GP and PN/DA
	Financing	*(-) Lack of time during consultation to discuss smoking*	GP and PN/DA
Inner setting	Compatibility	( +) AAC already known and applied in practice	GP and PN/DA
		(-) Did not use the method or used a different method	PN/DA
	Available resources	( +) A professional who offers counselling was employed in the practice during the study	GP and PN/DA
		(-) No counselling offered within the practice	GP and PN/DA
Characteristics of individuals	Motivation of innovation deliverers	(-) AAC sometimes feels inappropriate or pushy	GP
Implementation process	Engaging innovation deliverers	( +) More (knowledge of) possibilities for external smoking cessation counselling	GP and PN/DA
	Reflecting & Evaluating: Implementation	***(* +*) AAC carried out as planned, on individual level and/or practice level***	GP and PN/DA
		( ±) Attempts made to implement AAC but not completely successful yet	GP and PN/DA
		***(-) AAC insufficiently implemented***	GP and PN/DA

The ten most mentioned factors are printed in bold; ‘( +)’ indicates facilitators to implementation and ‘(-)’ indicates barriers to implementation

^a^Only mentioned by one participant

### Innovation

In the last questionnaire, 89% of the participants (*n* = 65) reported that applying AAC is more often easy than difficult for them. Also, 74% reported that applying AAC provides advantages, whereas only 9% reported that applying AAC provides disadvantages. Table 3 shows that participants most often mentioned as advantage that AAC makes it easier to discuss smoking cessation and provide a quit advice. As one PN wrote: *“[AAC] provides a nice and light start of the conversation about smoking cessation.”* Other important facilitators were related to the ‘complexity’ of the approach: AAC was mostly considered to be convenient and simple, and can be quickly applied. Some GPs, however, found the last step of ‘Connect’ to be a little more challenging and time-consuming. During the PTAMs, it was mentioned that most patients are not ready to be directly connected to a counsellor and first need to be motivated, and also that proactively referring patients costs extra time which GPs usually do not have.

### Outer setting

At baseline, 51% of the participants (*n* = 105) reported that the COVID-19 pandemic currently impacted smoking cessation care in their practice. After six months of participation (i.e., after the second PTAM), 34% of the participants (*n* = 56) reported that the COVID-19 pandemic had impacted smoking cessation care in their practice in the last three months. Table 3 shows that an important barrier to applying AAC and smoking cessation care in general experienced by participants was a lack of time or priority to address smoking. Several participants indicated that a lack of time in consultations is a structural problem in practice. One GP wrote: *“When patients consult me for something completely unrelated to smoking, there often isn’t enough time to start a conversation [about smoking].”* During the study, a lack of time or priority to discuss smoking with patients was also partly driven by the COVID-19 pandemic. A GP mentioned, when asked how the COVID-19 pandemic had impacted smoking cessation care: *“[Due to COVID-19] less attention could be paid to smoking cessation care because of all the other care which first needed to be caught up with.”* Here the GP refers to the lag in non-urgent care caused by the COVID-19 pandemic. Another GP wrote: “*Care has been very busy. That’s why I haven’t been able to ask [patients about smoking] as much as wanted.”*

Other COVID-19 related barriers were that consultations could not take place face-to-face anymore and that fewer patients consulted the practice. One GP wrote: *“Due to more telephone consultations, smoking is less easily brought up.”* Also, patients with smoking-related complaints or illnesses, such as asthma and chronic obstructive pulmonary disease (COPD), were seen less often during the COVID-19 pandemic. As one GP mentioned: *“We have seen fewer people in our consultations, especially fewer people with respiratory complaints. As a result, quitting smoking is less often discussed.”* Several participants also mentioned that smoking was less often discussed because fewer spirometry tests were performed.

Interestingly, while some participants perceived patients to be less motivated to quit due to COVID-19, other participants perceived patients to be more motivated to quit. Especially PNs/DAs mentioned that they received more requests for smoking cessation counselling from patients.

### Inner setting

With regard to the compatibility of AAC within practice, several PNs and DAs mentioned that they did not use the method or used a different method (see Table 3). One PN wrote: *“After 16 years of providing smoking cessation counselling, I have developed my own method which is difficult to change.”* Some GPs and PNs/DAs also mentioned that they already knew AAC and applied it in practice, and therefore the approach was not new to them. Within two practices, a professional who offers counselling was coincidentally employed during the study, which may have helped to implement AAC.

### Characteristics of individuals

After the first PTAM, most participants indicated that they felt able and motivated to apply the different steps of AAC in practice (*n* = 80). Table 4 shows that around 90% of the participants (completely) agreed that they felt able and motivated to ask patients about smoking and advise patients who smoke to quit, and around 80% of the participants (completely) agreed that they felt able and motivated to proactively refer patients who smoke. Only 60% of the participants (completely) agreed that they expected patients to react positively to ‘Ask’ and ‘Connect’, and 40% (completely) agreed that they expected patients who smoke to react positively to ‘Advise’. A chi-square test showed no significant differences between GPs and PNs/DAs. In the last questionnaire at the end of the study, the majority of the participants (i.e., 63%) agreed or completely agreed that Ask-Advise-Connect is a good method to reach patients with smoking cessation counselling (*n* = 65). A few GPs, however, felt that AAC is sometimes inappropriate or pushy (see Table 3). One GP wrote: *“As a general practitioner, I continue to find it difficult to ask every patient about smoking. For someone with a sore toe or vaginal complaints, that feels very inappropriate. With other complaints such as chest pain or dyspnoea this is much more logical.”*

**Table 4:** Proportion of participants who (completely) agreed with AAC-related statements, reported after the first PTAM (*n* = 80)

Statement	Total	GP	PN/DA
I feel **able** to ask patients about smoking. (Ask)	94%	98%	90%
I feel **motivated** to advise patients who smoke to quit. (Advise)	92%	95%	89%
I feel **motivated** to ask patients about smoking. (Ask)	91%	95%	87%
I feel **able** to advise patients who smoke to quit. (Advise)	90%	93%	87%
I feel **motivated** to proactively refer patients who smoke. (Connect)	84%	88%	79%
I feel **able** to proactively refer patients who smoke. (Connect)	80%	83%	76%
I **expect patients who smoke to react positively** when I proactively refer them. (Connect)	61%	60%	63%
I **expect patients to react positively** when I ask them about smoking. (Ask)	60%	60%	60%
I **expect patients who smoke to react positively** when I advise them to quit. (Advise)	40%	40%	39%

### Implementation process

As described before, participants made agreements on the delivery of AAC and reflected on these agreements during the PTAMs. Table 3 shows that the process of implementing AAC was perceived by many participants to have gone well, on an individual level and/or practice level. For example, one GP wrote: *“We now more actively ask [patients] about smoking, for example on the registration form for new patients.”* One PN wrote: *“It is nice that everyone in our practice is cooperating [in implementing AAC].”* It was, however, also often mentioned by participants that they had insufficiently implemented AAC according to plan. One GP wrote: *“[AAC] is not sufficiently ingrained in my consultation behaviour.”* Another GP wrote: *“I have difficulty with remembering to ask patients without smoking-related complaints whether they smoke.”* During the PTAMs, especially GPs indicated that they found it difficult to comply with the agreements, and that additional support would be helpful.

An important outcome of the implementation strategy (which was used to engage participants in implementing AAC), was that several participants indicated that they acquired more (knowledge of) possibilities for referring patients to external smoking cessation counselling (see Table 3). In fact, during the meetings three out of ten PTAM groups showed interest in working together with an external organisation offering group counselling. Eventually, this collaboration did not work out due to several reasons: in one PTAM group, the main healthcare insurance company did not reimburse counselling provided by an external organisation; another PTAM group failed to find a location for group counselling; in the third PTAM group, group counselling was organised once, but was cancelled a second time due to a lack of referrals.

## Discussion

This study aimed to identify which factors played a role in the implementation of AAC in Dutch general practice. A strength of this study is that we triangulated quantitative and qualitative findings in order to identify which factors played a role in the implementation of AAC. Another strength is that we included different types of healthcare providers who work in general practice, which allows us to make comparisons. In general, the AAC approach was received well by Dutch healthcare professionals in general practice: they viewed AAC as convenient, quick and simple, and felt that it made it easier for participants to discuss smoking cessation with patients who smoke and to give them a quit advice. Successful implementation of AAC was, however, hindered by several barriers, with the COVID-19 pandemic being the most important one. In particular a lack of time due to COVID-19 related priorities and consequent reduced priority to address smoking resulted in limited implementation of AAC. Important to note is that a lack of time to address smoking was already a problem for GPs before the COVID-19 pandemic [23]. Our findings show that the COVID-19 pandemic worsened this issue, despite the increased relevance of smoking cessation during the pandemic [3].

Not only was the implementation of AAC negatively affected by a lack of time and priority among healthcare providers, but also by the cancellation of consultations. Previous research found that patients refrained from visiting their GP because they did not want to burden their GP or feared getting infected with COVID-19 [24]. Also, as mentioned by our participants, and confirmed by previous research [25], especially patients with chronic lung diseases such as asthma and COPD were less often seen in practice during the pandemic. As patients with chronic lung diseases have an increased risk of developing severe COVID-19, general practices were advised at the beginning of the pandemic to not perform spirometry tests and to postpone the care for asthma and COPD patients [25]. With fewer options to provide regular care and fewer patients seen in practice, our participants had limited opportunities to discuss smoking with patients.

We noticed that more GPs compared to PNs/DAs, experienced difficulty implementing AAC. We suggest two possible explanations. First, most PNs and DAs in our study were already responsible for providing smoking cessation counselling in their practice (mostly for patients with chronic illnesses), even before the study started. They had their own procedures and systems in place to identify patients who smoke, provide a quit advice, and offer support. This may explain why only 7–9% of the PNs/DAs experienced barriers in applying AAC during the study, compared to 30–48% of the GPs, who were less used to provide smoking cessation support. Second, as observed in our own study and reported by other studies too [19, 23], many GPs only address smoking when they consider smoking to be relevant for the consultation (e.g., when a patient has smoking-related complaints). Previous research found that this is less of an issue for PNs, as most PNs find it important to address smoking regardless of the reason for the consultation [19], likely because the delivery of smoking cessation care is included as quality indicator in the care for chronically ill patients. Our results show that GPs have various reasons for not asking all of their patients about smoking, and that these reasons are found across different CFIR domains. GPs may not have enough time to address smoking during all consultations (domain ‘Outer setting’); GPs may not find it appropriate to ask about smoking if the patient has a complaint which the GP perceives to be unrelated to smoking (domain ‘Characteristics of individuals’); and some GPs simply forget to ask patients who have no smoking-related complaints about smoking, likely because there is no system which reminds them to do so (domain ‘Implementation process’).

With regard to the PNs and DAs, we found that several of them did not use the AAC method or used a different method. As mentioned in the results, most PNs and DAs were responsible for providing smoking cessation counselling in their practice, and thus were already quite experienced with delivering smoking cessation care. For these experienced practitioners the AAC method may have been too simple, implying that the method should perhaps be tailored according to the role and experience of the healthcare provider. For example, for experienced practitioners the AAC method may be extended to include more complicated skills, such as increasing the motivation of patients who smoke. As the GP has limited time to motivate patients to quit smoking, the PN and/or DA can play an important role in this.

### Implications

Considering that especially GPs experienced difficulty with implementing AAC, future implementation efforts should focus on providing additional support to GPs. For example, developing systems for building smoking cessation care into practice may help GPs to routinely carry out AAC. This may include incorporating an alert in the EHR which reminds GPs to ask about smoking, as well as a referral option in the EHR which automatically sends the patient’s contact details to a smoking cessation specialist who then may proactively contact the patient for an intake [11–14, 16, 17].

Also, e-health systems can help to reduce the workload of GPs, especially during stressful times. Research found that, as a result of the COVID-19 pandemic, e-health support interventions became more popular and more often used by Dutch healthcare professionals, and 48% of the GPs became more positive about options for digital contact with patients, for example through patient portals [26]. A digital patient portal offers patients access to their own medical data, and can also be used by patients to order repeat prescriptions and plan an appointment with their primary care provider. In 2021, 79% of Dutch general practices worked with digital patient portals, compared to only 42% in 2019 [26]. Future implementation efforts should consider using such digital patient portals to identify the smoking status of patients and motivate patients who smoke to quit, after which the GP receives an alert in the EHR to offer cessation support to identified patients who smoke during consultation.

Our findings also show that more is needed to make smoking cessation a priority within general practice, especially during stressful times in which the topic is easily put on the back burner. More attention could, for example, be paid to prevention and smoking cessation care during the training of medical students. Also, multimedia campaigns can be used to stimulate people to quit smoking and contact their GP office, which may put smoking cessation care higher on the agenda of general practices. Multimedia campaigns may also prevent patients from cancelling their appointments during future pandemics.

### Limitations

A few limitations of this study should be addressed. First, since we only collected qualitative data through open-ended questions in surveys, we were not able to ask further questions and thus our interpretations of the answers may be limited. The advantage of collecting data in this way, however, was that we were able to collect qualitative data from a large group of respondents. Second, not all participants completed all four surveys and we may have therefore missed certain views or experiences with regard to the implementation of AAC. Third, the study sample may not have been entirely representative of the larger population of primary care providers in general practice. Most PNs/DAs in our study already actively provided smoking cessation care, which is not necessarily the case for all PNs and DAs in the Netherlands. Also, AAC was not new to some participants, indicating an active interest of our participants in smoking cessation care. We expect the larger population of primary care providers to be less familiar with AAC, and as such, we expect that especially barriers with regard to its adoption will be encountered when AAC is implemented on a larger scale.

## Conclusions

Even though AAC was mostly positively received in general practice and primary care providers felt motivated and able to apply AAC, implementation turned out to be challenging, especially for GPs. Particularly external factors, such as a lack of time or priority to discuss smoking (due to COVID-19), negatively influenced implementation. Future efforts should focus on providing additional implementation support to GPs, for example with the use of e-health.

## Supplementary Information

**Additional file 1. Supplementary Table 1. **Differences in baseline characteristics between participants who completed atleast one follow-up questionnaire (i.e., Q2, Q3 or Q4) and participants who did not complete any follow-upquestionnaire (i.e., Q2, Q3 or Q4).

## Data Availability

The datasets generated and analysed during the current study are not publicly available, but are available from the corresponding author on reasonable request.

## Abbreviations

AAC: Ask-Advise-Connect
CFIR: Consolidated Framework for Implementation Research
DA: Doctor’s assistant
EHR: Electronic health record
GP: General practitioner
PN: Practice nurse
PTAM: Pharmaceutical Therapeutic Audit Meeting
