# Supplementary material for: Implementation of ask-advise-connect for smoking cessation in Dutch general practice during the COVID-19 pandemic: a mixed-methods evaluation using the CFIR framework
Source: Subst Abuse Treat Prev Policy. 2023 May 9;18:26. doi: 10.1186/s13011-023-00535-0 (PMC10169166; doi:10.1186/s13011-023-00535-0)
Supplement: Supplementary file 1 — Additional file 1. Supplementary Table 1. Differences in baseline characteristics between participants who completed atleast one follow-up questionnaire (i.e., Q2, Q3 or Q4) and participants who did not complete any follow-upquestionnaire (i.e., Q2, Q3 or Q4). [file 13011_2023_535_MOESM1_ESM.docx]

**Supplementary Table 1.** Differences in baseline characteristics between participants who completed at least one follow-up questionnaire (i.e., Q2, Q3 or Q4) and participants who did not complete any follow-up questionnaire (i.e., Q2, Q3 or Q4).

| **Variable** | **Category** | **Completed at least one follow-up questionnaire (n=89)**  n (%) / mean (SD) | **Did not complete any follow-up questionnaire (n=16)^a^**  n (%) / mean (SD) |
| --- | --- | --- | --- |
| Age |  | 45.3 (9.0) | 45.5 (10.8) |
| Gender | Male | 11 (12.4) | 8 (50.0)* |
|  | Female | 78 (87.6) | 8 (50.0) |
| Profession | General practitioner | 49 (55.1) | 14 (87.5)* |
|  | Practice nurse | 36 (40.4) | 0 (0.0) |
|  | Doctor’s assistant | 4 (4.5) | 2 (12.5) |
| Socioeconomic status of patients | Mostly low | 6 (6.8) | 0 (0.0) |
|  | Mostly middle | 30 (33.7) | 6 (37.5) |
|  | Mostly high | 4 (4.5) | 0 (0.0) |
|  | Mixed | 42 (47.2) | 10 (62.5) |
|  | Don’t know | 7 (7.9) | 0 (0.0) |
| Received training in smoking cessation care | Yes | 54 (60.7) | 5 (31.3)* |
|  | No | 35 (39.3) | 11 (68.8) |
| Applies smoking cessation guideline with patients who smoke | Never | 37 (41.6) | 7 (43.8) |
|  | Sometimes | 26 (29.2) | 7 (43.8) |
|  | Often | 17 (19.1) | 2 (12.5) |
|  | (Almost) always | 9 (10.1) | 0 (0.0) |
| Attention in practice for smoking cessation | Almost no attention | 3 (3.4) | 0 (0.0) |
|  | Some attention | 47 (52.8) | 11 (68.8) |
|  | A lot of attention | 39 (43.8) | 5 (31.3) |

^a^ Officially 17 participants did not complete any follow-up questionnaire, but one participant did not complete the baseline questionnaire and therefore only the characteristics of 16 participants are presented here.

*Chi-square test showed significant difference (*p*<0.05).
